# Nonprescription Products of Internet Retailers for the Prevention and Management of Herpes Zoster and Postherpetic Neuralgia: Analysis of Consumer Reviews on Amazon

**DOI:** 10.2196/24971

**Published:** 2021-02-08

**Authors:** Pengyi Zhu, Benjamin K P Woo

**Affiliations:** 1 Olive View-University of California Los Angeles Medical Center Sylmar, CA United States; 2 College of Osteopathic Medicine of the Pacific Western University of Health Sciences Pomona, CA United States

**Keywords:** Amazon, consumer preference, herpes zoster, postherpetic neuralgia, shingles, internet, customer, herpes, ingredients, treatment

## Abstract

**Background:**

Herpes zoster affects approximately 1 million people annually in the United States, with postherpetic neuralgia as the most common complication. The frequent prescription of opioids as the first-line medication for herpes zoster or postherpetic neuralgia contributes to the increasing health care costs of their treatment. Despite the advent of internet retailers providing alternative products for the prevention and management these conditions, there are limited studies on the availability, ingredients, and consumer preference for the products.

**Objective:**

This study used the internet retailer Amazon to determine the availability of products for the management of herpes zoster and postherpetic neuralgia, and assessed consumer preference based on listed ingredients.

**Methods:**

The internet retailer Amazon was used to perform a search for products related to “shingles” in September 2020. Top products sorted by reviews and ratings were determined to be either shingles-specific (including “shingles” in either the product title or description) or shingles-nonspecific. Analysis of price, rating, type of vehicle, and ingredients was performed. The types of vehicles, ingredients, and percentages of positive and negative reviews related to “shingles” of the product groups were analyzed with a two-tailed two-sample proportions *Z*-test to assess the difference between shingles-specific and shingles-nonspecific products. Statistical significance was judged at *P*<.05.

**Results:**

The top 131 products among over 3000 products retrieved were determined based on a rating of 4 or more stars after searching for the term “shingles” on Amazon. Forty-six of the 131 products (35.1%) were shingles-specific. Shingles-nonspecific products were more likely to have positive reviews mentioning “shingles” (*P*=.005). Vehicles, balms (*P*=.02), and salves (*P*=.04) were more likely to be shingles-specific, whereas tablets or capsules (*P*=.002) were more likely to be shingles-nonspecific. Among the ingredients analyzed, aloe vera was the top-ranked ingredient, included in 29 of the 131 total products (22.1%). Aloe vera (*P*=.01), lemon balm (*P<.*001), vitamin E (*P*=.03), and peppermint oil (*P*=.008) were more likely to be included in the shingles-specific products, whereas magnesium (*P*=.01) was more likely to be included in shingles-nonspecific products.

**Conclusions:**

There is an abundance of products and ingredients being used for the management and treatment of shingles with certain ingredients preferred by consumers. There is a discrepancy between approved ingredients and the ingredients preferred by consumers. Furthermore, there are insufficient studies on ingredients used by consumers on internet retailers such as Amazon, and future studies can focus on the effectiveness of popular ingredients to decrease misinformation on the internet.

## Introduction

Shingles, or herpes zoster (HZ), is caused by reactivation of latent varicella zoster virus (VZV) in the sensory neurons, typically after the primary infection of chicken pox in childhood [1,2]. Previous studies have shown that more than 95% of adults across North America and Europe are at risk for HZ, and approximately 1 million people are affected by HZ in the United States annually [2,3]. Stress, aging, illness, medication, or other causes of decreased immunity can cause the activation of dormant VZV that commonly affects the cervical, thoracic, and trigeminal nerves [3,4]. Acute herpes zoster (AHZ) manifests as a painful blistering rash along a dermatome, initially presenting as a maculopapular rash that develops into vesicles and pustules [4,5]. Postherpetic neuralgia (PHN) is the most common complication of HZ, which is defined as the persistence of acute HZ pain in a dermatomal distribution that lasts over 3 months [1,3,6,7]. PHN is due to damage to the nerves from an inflammatory response caused by viral replication within the nerve [8]. PHN can cause allodynia, hyperalgesia, anesthesia, and other sensory deficits of the affected dermatome [3,9]. Owing to the debilitating nature of PHN, patients seek medications to manage the pain.

The therapies for AHZ include antivirals, corticosteroids as adjunctive therapy, acetaminophen or nonsteroidal anti-inflammatory drugs (NSAIDs) for analgesia, and capsaicin or lidocaine as topical treatment [8]. For PHN, first-line medications include calcium channel blockers (gabapentin and pregabalin), tricyclic antidepressants, and a lidocaine patch [10]. Second-line treatments include opioid analgesics and a capsaicin patch or cream [10]. A previous study showed that opioids were commonly prescribed as the initial treatment for PHN, followed by gabapentin, prescription NSAIDs, lidocaine patch, pregabalin, tricyclic antidepressant, topical lidocaine, and capsaicin, respectively [3].

However, with the advent of internet information and shopping through internet retailers such as Amazon, patients can now easily access nonprescription remedies for the rashes and neuropathic pain caused by shingles or PHN. Amazon has recently been utilized as a data source in studies to analyze consumer perception and preference for health-related products [11-13]. Some natural treatment choices offered on internet retailers have been studied for either PHN or neuropathic pain, including vitamins and nutrients such as zinc, licorice, honey, aloe vera, and St John’s wort [14-16]. However, most herbal remedies such as St John’s wort have not been assessed in proper clinical trials or have not been proven to be useful [17].

Given the readily available nonprescription options that claim effectiveness for shingles and PHN pain, it is important to understand what products are available for patients and the efficacy of the ingredients in these products. In this study, we evaluated the products that are available and preferred by customers through analyzing the vehicle types, ingredients, and customer reviews of shingles-related products sold on Amazon. Finally, we sought to understand the different factors and ingredients of products used for shingles or PHN prevention and treatment.

## Methods

Amazon, the internet retailer, was accessed in September 2020 to search for products related to shingles. “Shingles” was used as the keyword to search in the “Health & Household” department of the website. The searches were screened for customer ratings of 4 stars and above. Products such as bandages or household items were excluded. Finally, only products that included the term “shingles” in the product title, description, Customer Reviews, or Customer Question and Answer were included in the final analysis. Product listings without mention of “shingles” in the product title, description, Customer Reviews, or Customer Question and Answer were excluded. For product listings with different quantities and sizes, only the listing with the most reviews was included. From the overall products, the average price, median price per unit, average ratings, average number of reviews, type of vehicle, and ingredients were determined. Several ingredients studied previously for shingles were analyzed in this study [14,17,18].

Two groups were derived from the original sample set. First, products that included the term “shingles” in either the product title or description were classified as shingles-specific products. Second, products that did not include the term “shingles” in either the product title or description, but included the term in either the Customer Reviews or the Customer Question and Answer sections were classified as shingles-nonspecific products. A two-tailed two-sample proportions *Z*-test was used to determine if a product specific to “shingles” was more likely to be a specific vehicle or to include certain ingredients. The numbers of positive and negative reviews in Customer Reviews including the term “shingles” were counted. The two-tailed two-sample proportions *Z*-test was also used to compare the means of percentages of positive and negative reviews among all reviews of products including “shingles” for shingles-specific and shingles-nonspecific products. A *P* value <.05 was considered to indicate a statistically significant difference.

## Results

Over 3000 results were populated on the Amazon internet retailer when searching for the key term “shingles.” There were 742 results in the “Health & Household” department that received a rating of 4 stars and above. Among the 131 top-rated products, there was a total of 215,225 reviews with a median of 698 reviews (range 24-16,523 reviews) and an average rating of 4.4 (out of 5) stars with a median of 4.4 (range 4-4.9 stars). Different types of vehicles for shingles were counted and categorized. Among the 131 total products, 44 (33.6%) were creams or lotions, 30 (22.9%) were tablets or capsules, 11 (8.4%) were gels, 10 (7.7%) were balms, 8 (6.1%) were bath products, 7 (5.3%) were ointments, 6 (4.6%) were oils, 5 (3.8%) were salves, and the remaining 10 products (7.6%) included powders, wipes, pump dispensers, liquids, and sprays. Among all reviews for the products that mentioned “shingles,” there was an average of 82.6% positive reviews and 10% negative reviews.

Among the 131 products, 46 (35.1%) products that included “shingles” in either the title or product description comprised shingles-specific products and 85 (64.9%) that did not include “shingles” in either the title or product description comprised the shingles-nonspecific products. Table 1 shows the top 10 most reviewed overall shingles products and Table 2 shows the top 10 most reviewed shingles-specific products. Comparison of the two tables reveals only three products among both the top 10 most reviewed overall products and the top 10 most reviewed shingles-specific products: Leven Rose Store’s Jojoba Oil, Emuaid’s EmuaidMAX Ointment, and Quantum Health’s Super Lysine.

**Table 1:** Top 10 most reviewed overall products.

Rank	Manufacturer	Product name	Number of reviews	Mean rating (out of 5)
1	Leven Rose Store	Jojoba Oil	16,523	4.7
2	Puriya	Mother of All Creams	11,139	4.3
3	Sun Essential Oils	Geranium	9046	4.2
4	Emuaid	EmuaidMAX Ointment	8974	4.0
5	Truremedy	Remedy Soap	8800	4.5
6	Mederma	Mederma Advanced Scar Gel	8063	4.2
7	Ramina	Natural Hemp Cream	6377	4.3
8	Puriya	Wonder Balm	6050	4.3
9	Quantum Health	Super Lysine	5875	4.6
10	NOW Foods	Double Strength L-Lysine	5607	4.7

**Table 2:** Top 10 most reviewed shingles-specific products.

Rank	Manufacturer	Product name	Number of reviews	Mean rating (out of 5)
1	Leven Rose Store	Jojoba Oil	16,523	4.7
2	Emuaid	EmuaidMAX Ointment	8974	4.0
3	Quantum Health	Super Lysine	5875	4.6
4	Blue Emu	Original Blue-Emu Super Strength	4723	4.5
5	Dermachange	Shingles	3254	4.2
6	Wild Naturals	Eczema & Psoriasis Cream	2896	4.1
7	Clearbody Organics	Hemp Relief Cream	2828	4.4
8	Era Organics	Relief	1885	4.0
9	Emuaid	Emuaid	1794	4.2
10	Frankincense & Myrrh	Neuropathy Rubbing Oil	1574	4.3

When analyzing customer reviews including “shingles” among the shingles-specific products, there was an average of 69.5% positive reviews and 10.1% negative reviews among the total shingles-related reviews per product. Among the shingles-nonspecific products, there was an average of 89.9% positive reviews and 10% negative reviews out of the total shingles-related reviews per product. Shingles-nonspecific products were more likely to have positive customer reviews mentioning “shingles” based on the *Z*-test analysis (*P*=.005), whereas there was no significant difference in negative customer reviews between shingles-specific and shingles-nonspecific groups (*P*=.99).

Out of the 46 shingles-specific products, 18 (39%) were creams or lotions, 7 (15%) were balms, 4 (9%) were oils, 4 (9%) were salves, 3 (7%) were tablets or capsules, 3 (7%) were gels, 3 (7%) were ointments, 1 (2%) was a bath product, and 3 (7%) were other products, including pump dispenser, spray, and liquid. Among the 85 shingles-nonspecific products, 27 (32%) were tablets or capsules, 26 (31%) were creams or lotions, 8 (9%) were gels, 7 (8%) were bath products, 4 (5%) were ointments, 3 (4%) were balms, 2 (2%) were oils, 1 (1%) was a salve, and 7 (8%) were other products, including powders, liquid, sprays, wipes, and solutions. Balms (*P*=.02) and salves (*P*=.04) were more likely to be shingles-specific, whereas tablets or capsules (*P*=.002) were more likely to be shingles-nonspecific.

Based on previous studies related to treatment for shingles, several ingredients were assessed from the products [14,17,18]. Among all 131 products, 29 (22.1%) contained aloe vera, 18 (13.7%) contained honey, 17 (13.0%) contained magnesium, 16 (12.2%) contained lemon balm, 14 (10.7%) contained L-lysine, 13 (9.9%) contained menthol, 11 (8.4%) contained lidocaine, 10 (7.6%) contained zinc, 9 (6.9%) contained oatmeal or oat straw extract, 7 (7.6%) contained St John’s wort, 6 (4.6%) contained licorice, 2 (1.5%) contained capsaicin, 2 (1.5%) contained Reishi mushroom, and 1 (0.8%) contained aspirin. Out the 46 shingles-specific products, 16 (34.8%) contained aloe vera, 10 (21.7%) contained honey, 1 (2.2%) contained magnesium, 12 (26.1%) contained lemon balm, 5 (10.9%) contained L-lysine, 3 (6.5%) contained menthol, 2 (4.3%) contained lidocaine, 4 (8.7%) contained zinc, 2 (4.3%) contained oatmeal or oat straw extract, 4 (8.7%) contained St John’s wort, 3 (6.5%) contained licorice, and none of the products contained capsaicin, Reishi mushroom, or aspirin. Among the 85 shingles-nonspecific products, 13 (15.3%) contained aloe vera, 8 (9.4%) contained honey, 16 (18.8%) contained magnesium, 4 (4.7%) contained lemon balm, 9 (10.6%) contained L-lysine, 10 (11.8%) contained menthol, 9 (10.6%) contained lidocaine, 6 (7.1%) contained zinc, 7 (8.2%) contained oatmeal or oat straw extract, 3 (3.5%) contained St John’s wort, 3 (3.5%) contained licorice, 2 (2.4%) contained capsaicin, 2 (2.4%) contained Reishi mushroom, and 1 (1.2%) contained aspirin. Among all ingredients, aloe vera (*P*=.01) and lemon balm (*P*<.001) were more likely to be included in shingles-specific products, whereas magnesium (*P*=.01) was more likely to be included in shingles-nonspecific products.

Vitamins were assessed individually [15,16,18]. Among all 131 products, 4 (3.1%) included vitamin A, 3 (2.3%) included vitamin B2, 4 (3.1%) included vitamin B6, 2 (1.5%) included vitamin B9, 2 (1.5%) included vitamin B12, 15 (11.5%) included vitamin C, 3 (2.3%) included vitamin D, and 28 (21.4%) included vitamin E. Among the 46 shingles-specific products, 8 (17%) included vitamin C and 15 (33%) included vitamin E, whereas no products included vitamins A, B2, B6, B9, B12, or D. Of the 85 shingles-nonspecific products, 4 (5%) included vitamin A, 3 (4%) included vitamin B2, 4 (4%) included vitamin B6, 2 (2%) included vitamin B9, 2 (2%) included vitamin B12, 7 (8%) included vitamin C, 3 (4%) included vitamin D, and 13 (15%) included vitamin E. Vitamin E was more likely to be used in shingles-specific products (*P*=.03).

Specific oils were assessed based off prior studies, including peppermint oil, geranium oil, and hemp oil [19-21]. Among all 131 products, 19 (14.5%) included peppermint oil, 5 (3.8%) included geranium oil, and 15 (11.5%) included hemp oil. Of the 46 shingles-specific products, 12 (26%) included peppermint oil, none included geranium oil, and 8 (17%) included hemp oil. Out the 85 shingles-nonspecific products, 7 (8%) included peppermint oil, 5 (6%) included geranium oil, and 7 (8%) included hemp oil. Peppermint oil was more likely to be included in shingles-specific products (*P*=.008).

## Discussion

### Principal Findings

With nearly 1 million cases of HZ diagnosed annually in the United States, HZ can affect up to 20% of individuals within their lifetimes [22,23]. HZ is a large health care burden, with opioids prescribed as the most common first-line treatment, further contributing to the already high health insurance cost of opioids [3]. Patients inflicted by PHN can spend 2-4 times more on health costs than patients with only HZ [24]. As such, internet retailers such as Amazon provide other options for the management and treatment of HZ or PHN that can provide relief for patients. However, with the wide variety of products and ingredients available, it can be difficult for patients to determine which products would be effective. This study showed that among the variety of vehicles available, Amazon customers preferred creams or lotions, which comprised 33.6% of the 131 products, with tablets or capsules coming in second comprising 22.9% of the 131 products. Tablets or capsules were more likely to be shingles-nonspecific products, whereas balms and salves were more likely to be shingles-specific products. This suggests a lack of consensus regarding products recommended for shingles and products that patients prefer.

Over 3000 products were retrieved when searching “shingles” on Amazon. “Shingles” was used as the keyword as it is a term more widely known in the general population. Among the products indicated for shingles treatment, there were also household cleaning products, which raises concern that such products are being suggested for shingles use on the internet retailer. Out of the 131 products, only 35.1% included products that specifically mentioned shingles in either the title or product description, and the majority (64.9%) of products did not. Furthermore, only 3 products from the top 10 most reviewed overall shingles products were within the top 10 most reviewed shingles-specific products. This highlights the discrepancy between products that are suggested for shingles use and products that are labeled for shingles use. When comparing customer reviews that mention shingles between shingles-nonspecific and shingles-specific products, shingles-nonspecific products were more likely to have more positive customer reviews (89.9%) compared with shingles-specific products (69.5%). This suggests that customer reviews play a larger role in product selection compared to a specified product indication for shingles. Interestingly, there was no significant difference in customer reviews of shingles-specific and shingles-nonspecific products, further compounding the importance of reviews in the consumer selection of products.

Various ingredients were analyzed that were previously studied in relation to shingles or PHN treatment. A 5% lidocaine patch and 0.075% capsaicin cream are among first- or second-line topical treatment options for PHN [8]. In our analysis of the 131 products, 8.4% contained lidocaine and 1.5% contained capsaicin. Of the shingles-specific products, only 4.3% contained lidocaine and none contained capsaicin, whereas of the shingles-nonspecific products, 10.6% contained lidocaine and 2.4% contained capsaicin. Lidocaine acts as a local anesthetic with a mechanism of action involving partial inhibition of voltage-gated calcium channels and reducing the discharge of activity in afferent pain receptors [25]. Lidocaine is considered a first-line therapy for PHN despite limited studies on its effectiveness [26,27]. The lidocaine products found in the Amazon products were between 4% and 5% in concentration; however, none of the top products recommended including lidocaine was a patch, which is recommended in the literature [8]. The products including lidocaine were creams, gels, or sprays, which suggests that lidocaine administered using these vehicles was preferred among customers instead of patches. Topical capsaicin is an activator of the TRPV1 channel of nociceptor nerve fibers, leading to an influx of calcium that decreases the function of nociceptor nerve fibers [25]. Studies have shown that a higher concentration of capsaicin was effective for PHN [28]. The concentration of capsaicin in the products found on Amazon was either 0.025% or 0.1%, whereas high-dose capsaicin products have a concentration of 8%. This may explain why capsaicin was not a highly preferred or recommended product as the smaller concentration only provides moderate relief for patients [25].

Other natural ingredients have been studied for their effects against HZ, such as licorice and Reishi mushroom [14]. Ingredients that have shown efficacy against herpes simplex virus (HSV) may also have a benefit for HZ, such as honey, aloe vera, and St John’s wort [14,29]. Licorice may be able to inactivate viral particles and was reported to show in vitro antiviral activity against VZV, although further studies are needed to evaluate its use as a topical agent [14]. Reishi mushroom was tested in a small clinical trial and a case study, demonstrating relief of pain [14]. Honey has shown faster healing times for patients with HSV infection, suggesting a possible benefit against HZ [14]. Aloe vera is a known ingredient for wound healing, and St John’s wort has shown antiviral activity against HSV-1 [14]. In this study, aloe vera was one of the top ingredients used among the 131 products, accounting for 22.1%, and is more likely to be used as an ingredient in shingles-specific products. This indicates that customers also prefer aloe vera for the treatment of shingles. Honey comprised 13.7% of all products, making it the second most likely included ingredient, suggesting that more studies should be performed to assess the efficacy of honey against HZ. St John’s wort, licorice, and Reishi mushroom were less commonly used as ingredients; however, more studies can reveal if these ingredients will be of benefit to patients with HZ or PHN. Lemon balm was also more likely to be included in shingles-specific products; despite few studies regarding the use of lemon specifically for this condition, it is likely preferred for shingles owing to its high vitamin C level.

Decreased immunity is a known risk factor for HZ and PHN, with nutritional deficiency being a major cause [18]. Vitamin and nutrient deficiencies, such as zinc and magnesium, and their effect on HZ have been studied to assess their efficacy as potential treatments [18,30,31]. Low vitamin C levels have been found to play a role in the development of herpes infection and PHN, with trials of intravenous vitamin C demonstrating efficacy in relieving pain [16]. Hypovitaminosis D has been associated with the development of neuropathic pain due to various mechanisms, including inflammatory processes and an increase of reactive oxygen species [15,32]. Vitamin B such as cobalamin (vitamin B12) was shown to be effective for painful neuropathies, and deficiency of folic acid (vitamin B9) causes peripheral neuropathy [33,34]. Vitamin E has also been shown to act as an analgesic in rat models with neuropathic pain [35]. Zinc deficiency has been shown to be a risk factor for PNH [30]. Magnesium has been found to block the N-methyl-D-aspartate receptor, which is associated with hypersensitivity [30,36]. Among the ingredients assessed, magnesium was more likely to be included in shingles-nonspecific products, whereas vitamin E was more likely to be included in shingles-specific products. Given the sparsity of studies on the efficacy of either magnesium or vitamin E, these results suggest that more studies are warranted for assessing these nutrients in the treatment of HZ and PHN. Vitamin C was the second-ranked nutrient among the 131 products, although it was not necessarily preferred in shingles-specific products despite studies showing its role in HZ and PHN. This discrepancy could be due to the lower concentration of vitamin C as either an oral supplement or within topical agents compared to a higher available systemic dose as an intravenous treatment.

Finally, certain oils were assessed, including peppermint oil, geranium oil, and hemp oil [19-21]. Peppermint oil was more likely to be used as an ingredient in shingles-specific products. The main ingredient of peppermint oil is menthol, which is commonly used for musculoskeletal pain [19]. The possible mechanism of action of peppermint oil is the inhibition of sensitized nociceptors [19]. However, few studies have assessed peppermint oil; thus, its common use in shingles-specific products warrants more studies on its effect on HZ and PHN. Geranium oil was found to relieve pain quickly in a small study [16]; however, it was not widely used in shingles products available on Amazon. Hemp oil was used a primary ingredient in several products (11.5% of 131 total products); however, few studies have focused on its effect for shingles. A small trial showed the effectiveness of a cannabinoid receptor agonist topical for PHN [21]. Given that it is commonly used, hemp oil should be further studied to better understand if it is effective for the treatment of shingles.

### Limitations

The main limitation of the study is that not all of the products related to “shingles” from Amazon were included in the analysis since there was over 3000 products found. Only the top 131 products were included with products receiving a rating of less than 4 stars excluded. Furthermore, products that did not include “shingles” in either the product name, description, or mentioned in the customer reviews were excluded. This excluded products that were suggested by the internet retailer algorithm but may not have been suggested by the manufacturer or customers to be used for shingles. Another limitation was that only “shingles” was used as the keyword to narrow down the search based on what the general populace would search. Searching for “postherpetic neuralgia” and “herpes zoster” could potentially produce more results. Furthermore, we did not analyze all of the active ingredients used by all of the products analyzed. Finally, we did not compare ingredients of the products with the amount of positive and negative reviews related to “shingles” that the product received. This comparison would allow for further analysis of whether specific ingredients were perceived to be effective to the general population.

### Conclusion

Our analysis of “shingles” products on the internet retailer Amazon demonstrated an abundance of products and available ingredients used for shingles treatment. Although there are already available treatments that are approved for the management of AHZ and PHN, because these are conditions that are typically managed by several treatments, understanding over-the-counter management would benefit patients. Using Amazon to understand what is available to and preferred by customers can allow us to assess which ingredients require further studies to better educate our patients on what would be effective for AHZ and PHN and to target potential misinformation online.

## Abbreviations

AHZ: acute herpes zoster
HSV: herpes simplex virus
HZ: herpes zoster
NSAIDs: nonsteroidal anti-inflammatory drugs
PHN: postherpetic neuralgia
VZV: varicella zoster virus
